# Psychological profile and heart rate variability in adults with self-perceived chronic stress: secondary analysis from a clinical trial

**DOI:** 10.3389/fphys.2026.1753526

**Published:** 2026-07-16

**Authors:** Ana Isabel Pérez Alcalde, María-José Giménez, Cecilia Estrada Barranco, Francisco J. Fernández-Rodríguez, Marta de la Plaza, María García-Arrabé, Beatriz Ruiz-Ruiz

**Affiliations:** Universidad Europea de Madrid, Faculty of Medicine, Health and Sports, Department of Physiotherapy, Madrid, Spain

**Keywords:** autonomic regulation, chronic stress, conscientiousness, heart rate variability (HRV), neurovisceral integration, psychological inflexibility, self-perceived stress, sex differences

## Abstract

**Background:**

Chronic stress is a major public health issue linked to psychological vulnerability and autonomic dysregulation. While heart rate variability (HRV) is widely recognized as a biomarker of stress adaptability, few studies have characterized the psychophysiological profile of individuals who self-identify as chronically stressed. Understanding this relationship could clarify the integration between subjective perception and autonomic control mechanisms.

**Methods:**

A cross-sectional study was conducted with 54 adults reporting chronic self-perceived stress for at least 6 months. Psychological assessment included validated Spanish versions of the Perceived Stress Scale (PSS-4), State–Trait Anxiety Inventory (STAI-6), Zung Self-Rating Depression Scale (SDS), Acceptance and Action Questionnaire (AAQ-II), UCLA Loneliness Scale, and Ten-Item Personality Inventory (TIPI). HRV was recorded using a Polar H10 sensor in 2 daily 5-min supine measurements (morning and evening) over 7 days. Time, frequency, and nonlinear-domain parameters were analyzed according to the Task Force standards.

**Results:**

Participants presented high levels of anxiety, depression, and psychological inflexibility, together with moderately reduced HRV indices. No significant differences were found between levels of perceived stress. Higher conscientiousness showed modest positive correlations with SDNN, RMSSD, pNN50, and HRV triangular index, while sex differences were observed in frequency-domain HRV parameters. Overall, HRV was not clearly differentiated across perceived stress levels.

**Conclusion:**

Adults with chronic self-perceived stress exhibit a psychophysiological pattern characterized by emotional vulnerability, cognitive overcontrol, and reduced autonomic flexibility. This state reflects latent regulatory vulnerability rather than an overt autonomic failure. HRV assessment combined with psychological profiling may enhance early identification of individuals at risk and support integrative approaches for stress regulation.

## Introduction

Chronic stress is one of the leading public health problems worldwide, with significant repercussions for physical, psychological, and social health. The World Health Organization (WHO) defines it as a reaction of the organism to demands that exceed perceived coping resources and recognizes it as a highly prevalent phenomenon (Health, 2022). It is estimated that more than one billion people worldwide experience it (Vigo et al., 2016), and recent international surveys show that up to 40% of adults report high levels of stress in their daily lives (Gallup, 2022). This impact goes beyond the individual, contributing to increased healthcare costs, work absenteeism, and reduced quality of life, positioning chronic stress as a global health priority (Franklin et al., 2021).

Although stress initially serves an adaptive function, its persistence over time has been linked to multiple negative consequences, including cardiovascular disease, sleep disturbances, metabolic disorders, and an increased risk of anxiety and depression, as well as unhealthy behaviors such as substance use and physical inactivity (Cohen et al., 2007; McEwen, 2007; Kiecolt-Glaser et al., 2017; Madison et al., 2022; Santos et al., 2022). Beyond these well-documented health outcomes, a growing body of research indicates that both exposure to stressful life events and sustained elevations in perceived stress are associated with adverse effects on brain health and cognitive functioning across the lifespan. Systematic reviews have shown that stressful life events may contribute to long-term alterations in neuroendocrine regulation, neural integrity, and increased vulnerability to cognitive decline (Giannouli, 2025). In parallel, empirical evidence suggests that higher levels of perceived stress are associated with poorer global cognitive performance and difficulties in instrumental activities of daily living, even among non-clinical adult populations (Giannouli and Tsolaki, 2023).

Contemporary models, such as Porges’ Polyvagal Theory and the Neurovisceral Integration Model proposed by Thayer and Lane, highlight the role of the vagus nerve and the autonomic nervous system (ANS) in the regulation of emotional and physiological responses (Benarroch, 1993; Porges, 2011; Thayer et al., 2011; Smith et al., 2017).

In this context, the self-perception of stress acquires particular relevance. The subjective appraisal an individual makes regarding their own stress state constitutes a clinical indicator of psychological distress and is accompanied by symptoms such as anxiety, depression, loneliness, or cognitive rigidity (Altungy et al., 2023; Calderón-García et al., 2024). Validated instruments are used to characterize these dimensions and accurately describe the psychological profile of individuals who consider themselves chronically stressed, such as the Perceived Stress Scale (Warttig et al., 2013), the State–Trait Anxiety Inventory (Marteau and B, 2020), the Zung Self-Rating Depression Scale (Zung, 1965), the Acceptance and Action Questionnaire (Ruiz et al., 2016), the UCLA Loneliness Scale (Hughes et al., 2004), and brief personality trait measures such as the NEO-FFI (Manga et al., 2004) or the Ten-Item Personality Inventory (Thørrisen and Sadeghi, 2023).

Stress perception and physiological regulation are not independent processes but interconnected dimensions of a complex psychobiological response, which may show different levels of correspondence across individuals (Smith et al., 2017; Porges, 2022).

However, self-perception is not limited to the subjective domain. Numerous studies have shown that high levels of perceived stress are associated with objective physiological changes, particularly in heart rate variability (HRV) (Malik, 1996; Shaffer and Ginsberg, 2017; Veloza et al., 2019; Shaffer and Meehan, 2020). HRV reflects the dynamic interaction between the sympathetic and parasympathetic branches of the autonomic nervous system and constitutes a sensitive biomarker of the organism’s adaptive capacity (Lehrer, 2013; Smith et al., 2017; Massaro and Pecchia, 2019). High HRV indicates physiological flexibility and adequate autonomic balance, whereas reduced values have been associated with diminished regulatory capacity and with states of psychological and medical vulnerability (Teodorescu and Nanea, 2014; Smith et al., 2017). In individuals who consider themselves chronically stressed, reductions in vagal parameters such as RMSSD, NN50, and pNN50 have been observed, suggesting impaired parasympathetic modulation (Schneider and Schwerdtfeger, 2020; Calderón-García et al., 2024).

Despite the growing use of HRV as a biomarker in clinical research and digital monitoring technologies (e.g., wearable devices) (Hayano and Yuda, 2019; Massaro and Pecchia, 2019), a knowledge gap remains: studies that specifically describe the baseline psychophysiological profile of individuals who self-identify as chronically stressed are scarce (Schubert et al., 2009; Sharma and Gedeon, 2012; Chand et al., 2024). This gap limits the accurate interpretation of HRV in clinical and experimental contexts, as normative values may not be representative for this population (Nunan et al., 2010; Lee, 2022).

Given the relationship between self-perceived stress and objective parameters of autonomic regulation, it is essential to specifically characterize the psychophysiological profile of individuals who consider themselves chronically stressed (Schubert et al., 2009; Clemente-Suárez and Ruisoto-Palomera, 2020; Calderón-García et al., 2024). Such knowledge allows for a better understanding of the convergence between subjective experience and physiological markers and has important implications for research and clinical practice (Smith et al., 2017; Rodríguez-Jiménez et al., 2022; Rusanov et al., 2022). In particular, in studies using HRV as a biomarker, baseline HRV values should be interpreted with consideration of chronic stress self-perception, as they may differ significantly depending on this factor. This underscores the need to establish normative descriptions in this population and provides a reference framework for future research and interventions aimed at promoting psychophysiological well-being (Nunan et al., 2010; Schneider and Schwerdtfeger, 2020; Chand et al., 2024).

## Methods

### Study design

Participants were recruited between January and March 2024 from employees of the European University of Madrid (Spain) through informational posters strategically placed across university facilities. Eligibility criteria included adults aged 18 years or older who reported experiencing self-perceived stress for a minimum duration of six months. Exclusion criteria comprised the consumption of tea, caffeine, energy drinks, alcohol, or tobacco within the two hours preceding assessment; the presence of neck pain or clinically significant headache; carotid sinus syndrome; pregnancy; recent cervical or cardiac surgery; recent major trauma; cancer; neurological conditions affecting muscle tone; chronic medical conditions such as diabetes mellitus or hypertension; use of beta-blockers; and the presence of arrhythmias or other cardiovascular diseases (Pérez-Alcalde et al., 2024).

As this is a secondary analysis, the inclusion and exclusion criteria of the original randomized controlled trial—designed to assess the effects of a neurodynamic intervention in adults with self-perceived chronic stress—defined a clinically homogeneous sample and did not introduce additional selection bias beyond the target population of interest; therefore, the psychological profile observed reflects the expected characteristics of this population (Pérez-Alcalde et al., 2024).

The analyses presented here focus on preliminary data collected using validated Spanish versions of psychological instruments during the week prior to the participants’ physiological assessment. For these analyses, participants were considered as a single group, without regard to randomization, since the data were collected before the application of the interventions compared in the clinical trial.

### Procedure

#### Psychological assessment

During the week prior to the physiological evaluation, participants completed a battery of validated psychological instruments in their Spanish versions:

- The Perceived Stress Scale (PSS-4), a four-item measure with a five-point Likert response format, was used to estimate subjective stress perception over the past month, the following cut-off points were used to categorize the measures: PSS-4 scores were classified as *low* (≤4), *moderate* (5–8), *high* (9–12), and *very high* (>12) (Warttig et al., 2013).
- To assess anxiety levels, the six-item abbreviated version of the State–Trait Anxiety Inventory (STAI-6) was used, designed to measure state and trait anxiety in a rapid and reliable manner. For the STAI-6, scores <50 were classified as *normal*, whereas values ≥50 were considered *high* (Marteau and B, 2020).
- For descriptive purposes only, participants were categorized according to the established cut-off scores for the PSS-4 and STAI-6 scales. However, all inferential statistical analyses were performed using the original continuous scores.
- Depressive symptoms were assessed using the Zung Self-Rating Depression Scale (SDS), a 20-item instrument that evaluates affective, somatic, cognitive, and psychomotor aspects of depression (Zung, 1965).
- Perceived loneliness was measured using the abbreviated three-item version of the UCLA Loneliness Scale (Hughes et al., 2004).
- Psychological flexibility was assessed using the Acceptance and Action Questionnaire (AAQ-II), a seven-item measure scored on a 0–7 Likert scale, where higher values indicate greater experiential avoidance and inflexibility (Ruiz et al., 2016).
- Personality traits were assessed using the Ten-Item Personality Inventory (TIPI), a brief ten-item questionnaire that provides a rapid assessment of the main dimensions of the Five-Factor Model (Thørrisen and Sadeghi, 2023).

#### Physiological assessment

To determine the physiological profile, heart rate variability (HRV) was recorded using a Polar H10 chest strap (Polar Electro^®^, Kempele, Finland). Measurements were taken in the supine position: one in the morning, immediately after nocturnal rest, and another in the evening, just before going to bed. Participants were instructed to wear the chest strap for approximately 7 minutes per recording to ensure the availability of a continuous and stable 5-minute segment for HRV analysis, in line with Task Force recommendations for short-term recordings (Malik, 1996; Goroso et al., 2021). Participants were also instructed to maintain consistent recording conditions throughout the monitoring period and to avoid caffeine, tea, energy drinks, alcohol, and tobacco before the evening recordings.

HRV analysis was conducted in accordance with the recommendations of the Task Force of the European Society of Cardiology and the North American Society of Pacing and Electrophysiology (Malik, 1996), which establish standard criteria for short-term HRV recording and analysis. RR intervals were recorded using a Polar H10 sensor operating at a sampling frequency of 135 Hz, which falls within the range considered adequate for short-term HRV analysis.

Given the home-based nature of the protocol, all recordings were screened after data acquisition for signal quality and artifact burden. Artifact percentage was automatically computed by Kubios HRV Scientific during segment selection. In accordance with Task Force recommendations, recordings presenting an artifact rate ≥5% were excluded, and no imputation of missing data was performed. For each participant, HRV parameters were calculated as the mean of all valid recordings over the monitoring period, providing a stable estimate of basal autonomic function.

The HRV variables selected for analysis included time-domain, frequency-domain, and nonlinear parameters. In the time domain, the following were examined: mean interbeat interval (Mean RR), standard deviation of NN intervals (SDNN), mean heart rate (Mean HR), minimum heart rate (Min HR), maximum heart rate (Max HR), the difference between them (Dif HR), the root mean square of successive differences (RMSSD), the number of NN intervals differing by more than 50 ms (NN50), the corresponding percentage (pNN50), and the HRV triangular index.

In the frequency domain, low-frequency power (LF n.u.), high-frequency power (HF n.u.), and the LF/HF ratio were included. Finally, nonlinear analysis considered parameters derived from the Poincaré plot: short-term standard deviation (SD1), long-term standard deviation (SD2), and their ratio (SD2/SD1) (Shaffer and Ginsberg, 2017; Shaffer and Meehan, 2020).

### Statistical analysis

Sample size estimation was performed with Granmo version 8.0, taking heart rate variability (HRV) as the primary outcome and based on data reported by Hernando et al (Hernando et al., 2016). An alpha level of 0.05 and statistical power of 80% were specified for a two-sided test. Under these assumptions, 31 participants per group were required to detect an HRV difference of 0.12, assuming a pooled standard deviation of 0.15. A projected attrition rate of 20% was also incorporated into the calculation. Although the sample size was calculated for the original randomized controlled trial, the present study includes the full sample and constitutes a secondary descriptive analysis focused on the baseline psychophysiological and psychological characteristics of adults with self-perceived chronic stress.

Statistical analysis was performed using Jamovi software (version 2.3.28). Quantitative variables were described using the mean and standard deviation, or the median and interquartile range (P_25_, P_75_) when necessary, given the dispersion of the data. The normality of the variables was assessed using the Shapiro–Wilk test and Q-Q plots. Given the non-normal nature of the distribution of the variables, the Mann–Whitney U test or the Kruskal-Wallis test were used for comparisons between groups. Correlations between HRV variables and psychological variables, as well as between the psychological variables themselves, were investigated using Spearman’s correlation coefficient. Correlation coefficients were classified as negligible (ρ < 0.3), low (ρ ≥ 0.3 and < 0.5), moderate (ρ ≥ 0.5 and < 0.7), high (ρ ≥ 0.7 and < 0.9), and very high (ρ ≥ 0.9) (Mukaka, 2012). Internal consistency of the psychometric scales was examined in the present sample by calculating both Cronbach’s alpha and McDonald’s omega coefficients.

The results were considered significant with a p-value ≤ 0.05.

## Results

The study included 54 individuals, of which 41 (75.9%) were females. Mean age was 44.9 ± 11.5 years (41.39 ± 9.74 for males and 46.07 ± 11.93 for females, p=0.188). Participants reported sleeping an average of 6.87 ± 0.80 h per day (range: 5–9 h), watching television for a median of 1 h per day (range: 0–4.5 h), and spending a median of 1 h per day on social media (range: 0–5 h), with no sex-related differences observed for any of these variables. **Table 1** shows the HRV parameters and psychological variables of the participants distributed by sex. Statistically significant differences were observed in some frequency-domain HRV variables and in neuroticism, with higher neuroticism scores in females (p ≤ 0.001).

**Table 1:** Heart rate variability and psychological variables of participants distributed by gender.

Parameter	Males (n=13)	Females (n=41)	p
Mean RR (bpm)	980.14 (832.58, 1015.92)	905.30 (845.97, 943.08)	0.764
SDNN (ms)	35.67 (32.74, 46.62)	33.64 (26.80, 46.44)	0.411
Mean HR (bpm)	63.07 (59.30, 72.78)	66.805 (63.13, 71.72)	0.795
Min HR (bpm)	56.53 (54.20, 65.67)	61.08 (56.97, 63.49)	0.484
Diff. HR (bpm)	19.88 (14.93, 21.18)	17.59 (13.91, 20.18)	0.102
Max HR (bpm)	77.21 (71.52, 84.45)	77.88 (71.01, 82.89)	0.842
RMSSD (ms)	35.31 (23.20 43.84)	34.47 (23.79, 46.66)	0.857
NN50 (number of NN intervals)	35.57 (13.21, 52.15)	37.69 (16.29, 50.82)	0.968
pNN50 (%)	0.15 (0.04, 0.22)	0, 12 (0.04, 0.22)	0.936
HRV triangular index	8.98 (7.75, 10.87)	8.43 (7.10, 10.26)	0.378
LF (n.u.)	73.25 (58.86, 80.10)	63.28 (51.01, 66.97)	**0.022**
HF (n.u.)	26.67 (20.75, 41.13)	36.71 (32.98, 48.98)	**0.029**
LF/HF	3.69 (2.08, 4.66)	2.23 (1.26, 2.74)	**0.011**
SD1(ms)	26.48 (21.68, 31.05)	23.07 (15.03, 28.29)	0.400
SD2 (ms)	43.65 (36.24, 59.05)	43.90 (33.42, 50.22)	0.704
SD2/SD1	2.25 (1.90, 2.32)	2.03 (1.87, 2.41)	0.857
Extraversion	6.23 ± 1.36	5.76 ± 1.34	0.187
Agreeableness	6.46 ± 2.11	6.90 ± 1.50	0.299
Conscientiousness	7.92 ± 1.50	8.02 ± 1.80	0.665
Neuroticism	5.15 ± 1.72	7.32 ± 1.85	**<0.001**
Openness	7.54 ± 1.39	7.88 ± 1.69	0.374
Anxiety (STAI-6)	14.08 ± 4.75	15.49 ± 3.05	0.209
Psychological inflexibility (AAQII)	20.08 ± 8.65	23.98 ± 8.97	0.232
Loneliness (UCLA)	6.62 ± 1.56	6.81 ± 1.17	0.925
Stress (PSS-4)	5.77 ± 3.30	6.93 ± 3.33	0.203
Depression (SDS)	46.39 ± 5.52	48.07 ± 5.85	0.378

Data are expressed as mean ± standard deviation or median (P_25_, P_75_). Values in bold indicate statistically significant differences. Mean RR, Mean interbeat interval; SDNN, Standard Deviation of NN Intervals; Mean HR, mean Heart Rate; Min HR, minimal Heart Rate; Diff HR, Heart Rate Difference; Max HR, maximum interbeat interval; RMSSD, Root Mean Square of Successive Differences; NN50, Number of NN50 Intervals; pNN50, Percentage of NN50 Intervals; LF, Normalized low-frequency power; HF, Normalized high-frequency power; SD1, Short-term standard deviation; SD2, Long-term standard deviation. PSS-4, Perceived Stress Scale; STAI-6, State–Trait Anxiety Inventory; AAQ-II, Acceptance and Action Questionnaire; SDS, Zung Self-Rating Depression Scale. bpm, Beats per minute; ms, milliseconds; %, percentage; n.u., normalized units.

**Table 2** shows the HRV parameters and psychological variables of the participants distributed by reported level of stress. Statistically significant differences (p < 0.001) were found in the mean PSS-4 scores across the different levels, with increasing values as the level increased: 3.11 ± 1.18, 6.82 ± 1.05, 10.00 ± 1.18, and 14.33 ± 0.58 from low to very high. No significant differences were found between the groups in age, daily sleep hours, time spent watching TV, or time spent on social media. As shown in the Table, anxiety, psychological inflexibility, and depression exhibited a significant and progressive increase in scores from the low-level stress group to the very high-level stress group.

**Table 2:** Heart rate variability and psychological variables of participants distributed by level of stress measured by the PSS-4 scale.

Parameter	Low (n=18)	Moderate (n=22)	High (n=11)	Very high (n=3)	p
Mean RR (bpm)	872.46 (820.07, 957.69)	918.91 (833.57, 1003.03)	905.75 (852.84, 921.29)	1015.92 (935.01, 1020.47)	0.629
SDNN (ms)	34.9 (27.22, 45.66)	39.89 (29.81, 48.21)	32.9 (28.92, 39.18)	30.28 (24.64, 31.89)	0.496
Mean HR (bpm)	69.08 (63.09, 73.53)	65.64 (60.12, 72.63)	67.32 (62.81, 71.13)	59.30 (58.99, 65.02)	0.619
Min HR (bpm)	62.32 (57.10, 65.59)	58.97 (54.60, 65.21)	61.27 (59.61, 63.00)	55.08 (55.07, 60.28)	0.638
Diff. HR (bpm)	17.68 (15.18, 20.70)	19.84 (15.24, 21.02)	17.51 (13.17, 19.16)	13.55 (12.95, 13.77)	0.131
Max HR (bpm)	80.15 (73.01, 86.00)	77.94 (72.25, 84.53)	77.85 (70.89, 82.38)	69.08 (68.85, 73.45)	0.309
RMSSD (ms)	32.27 (21.33, 49.21)	36.01 (25.23, 44.93)	34.80 (25.93, 40.00)	34.47 (25.08, 38.15)	0.931
NN50 (number of NN intervals)	34.41 (12.19, 57.12)	39.49 (16.82, 52.05)	43.08 (20.93, 47.70)	18.78 (10.47, 42.43)	0.972
pNN50 (%)	0.11 (0.03, 0.28)	0.15 (0.04, 0.18)	0.14 (0.07, 0.19)	0.07 (0.04, 0.15)	0.923
HRV triangular index	8.38 (7.12, 10.84)	9.17 (7.63, 10.81)	8.43 (7.89, 10.06)	6.79 (6.12, 7.47)	0.388
LF (n.u.)	66.50 (57.21, 77.18)	64.03 (51.47, 71.84)	63.28 (53.61, 66.51)	53.91 (48.02, 63.05)	0.690
HF (n.u.)	33.47 (22.81, 40.39)	35.94 (28.10, 48.51)	36.70 (33.45, 46.37)	46.04 (36.89, 51.89)	0.686
LF/HF	3.04 (2.01, 4.43)	2.27 (1.29, 3.38)	2.12 (1.58, 2.43)	1.49 (1.18, 2.34)	0.409
SD1(ms)	22.53 (14.45, 26.53)	25.81 (18.24, 34.82)	23.60 (16.64, 24.65)	12.15 (11.18, 20.89)	0.291
SD2 (ms)	46.97 (32.96, 54.39)	46.47 (37.27, 58.81)	41.63 (31.04, 46.52)	33.84 (27.48, 35.19)	0.208
SD2/SD1	2.23 (2.05, 2.46)	1.94 (1.72, 2.30)	1.98 (1.79, 2.22)	2.27 (1.77, 2.97)	0158
Extraversion	6.33 ± 1.41	5.86 ± 1.13	5.18 ± 1.47	5.67 ± 1.53	0.316
Agreeableness	6.61 ± 1.33	7.18 ± 1.65	6.27 ± 1.90	7.00 ± 2.65	0.533
Conscientiousness	8.22 ± 2.05	8.00 ± 1.41	7.73 ± 1.95	7.67 ± 1.15	0.668
Neuroticism	6.33 ± 2.40	6.99 ± 1.77	7.36 ± 1.86	6.67 ± 2.52	0.722
Openness	7.55 ± 1.42	8.32 ± 1.55	7.09 ± 1.81	8.00 ± 2.00	0.233
Anxiety (STAI-6)	12.50 ± 3.03	15.59 ± 2.46	16.73 ± 2.72	22.00 ± 1.73	**<.001**
Psychological inflexibility (AAQII)	16.67 ± 5.68	24.23 ± 7.16	26.73 ± 9.18	39.00 ± 7.81	**<.001**
Loneliness (UCLA)	7.06 ± 1.06	6.68 ± 1.29	6.82 ± 1.47	5.33 ± 0.58	0.115
Depression (Zung scale)	44.22 ± 4.31	47.41 ± 3.94	50.00 ± 4.69	61.67 ± 4.51	**<.001**

Data are expressed as mean ± standard deviation or median (P_25_, P_75_). Values in bold indicate statistically significant differences. Mean RR, Mean interbeat interval; SDNN, Standard Deviation of NN Intervals; Mean HR, mean Heart Rate; Min HR, minimal Heart Rate; Diff HR, Heart Rate Difference; Max HR, maximum interbeat interval; RMSSD, Root Mean Square of Successive Differences; NN50, Number of NN50 Intervals; pNN50, Percentage of NN50 Intervals; LF, Normalized low-frequency power; HF, Normalized high-frequency power; SD1, Short-term standard deviation; SD2, Long-term standard deviation. PSS-4; Perceived Stress Scale; STAI-6, State– ,Trait Anxiety Inventory; AAQ-II, Acceptance and Action Questionnaire; SDS, Zung Self-Rating Depression Scale. bpm, Beats per minute; ms, milliseconds; %, percentage; n.u., normalized units.

**Table 3** shows the HRV parameters and psychological variables of the participants distributed by anxiety level. No significant differences were found between groups in HRV parameters, age, daily sleep hours, time spent watching TV, or time spent on social media. Neuroticism, psychological inflexibility, perceived stress, and depression scores were significantly higher among participants with high anxiety.

**Table 3:** Heart rate variability and psychological variables of participants distributed by level of anxiety measured by the STAI-6 scale.

Parameter	Normal (n=23)	High (n=31)	p
Mean RR (bpm)	899.56 (841.43, 965.90)	905.75 (837.80, 998.77)	0.716
SDNN (ms)	34.99 (31.10, 48.58)	33.50 (27.65, 44.35)	0.445
Mean HR (bpm)	67.21 (62.36, 71.87)	66.40 (60.39, 72.20)	0.890
Min HR (bpm)	60.89 (56.51, 64.24)	60.69 (55.44, 64.36)	0.917
Diff. HR (bpm)	18.75 (14.88, 21.22)	17.62 (13.95, 19.88)	0.281
Max HR (bpm)	79.81 (73.24, 83.97)	77.47 (71.26, 83.13)	0.543
RMSSD (ms)	33.33 (23.59, 47.90)	34.80 (22.21, 42.83)	0.487
NN50 (number of NN intervals)	35.57 (17.03, 48.64)	37.80 (11.91, 59.29)	0.931
pNN50 (%)	0.13 (0.05, 0, 24)	0, 13 (0.04, 0.19)	0.375
HRV triangular index	8.93 (7.42, 10.97)	8.43 (6.87, 10.27)	0.445
LF (n.u.)	64.99 (53.04, 78.64)	64.02 (52.30, 68.08)	0.487
HF (n.u.)	34.98 (21.31, 46.74)	35.95 (31.90, 47.68)	0.567
LF/HF	2.74 (1.21, 4.56)	2.24 (1.43, 2.93)	0.445
SD1(ms)	25.97 (20.71, 31.58)	21.70 (13.17, 28.32)	0.180
SD2 (ms)	47.61 (37.77, 59.24)	39.02 (33.09, 48.49)	0.097
SD2/SD1	2.03 (1.70, 2.37)	2.12 (1.90, 2.38)	0.543
Extraversion	5.74 ± 1.29	5.97 ± 1.40	0.445
Agreeableness	6.70 ± 1.52	6.87 ± 1.77	0.736
Conscientiousness	8.22 ± 1.57	7.84 ± 1.83	0.510
Neuroticism	5.70 ± 2.01	7.61 ± 1.65	**<0.001**
Openness	7.87 ± 1.39	7.74 ± 1.79	0.886
Psychological inflexibility (AAQII)	19.26 ± 7.66	25.84 ± 8.95	**0.013**
Loneliness (UCLA)	6.87 ± 1.42	6.68 ± 1.14	0.293
Stress (PSS-4)	5.00 ± 2.59	7.87 ± 3.32	**0.003**
Depression (Zung scale)	45.52 ± 4.19	49.26 ± 6.31	**0.018**

Data are expressed as mean ± standard deviation or median (P_25_, P_75_). Values in bold indicate statistically significant differences. Mean RR, Mean interbeat interval; SDNN, Standard Deviation of NN Intervals; Mean HR, mean Heart Rate; Min HR, minimal Heart Rate; Diff HR, Heart Rate Difference; Max HR, maximum interbeat interval; RMSSD, Root Mean Square of Successive Differences; NN50, Number of NN50 Intervals; pNN50, Percentage of NN50 Intervals; LF, Normalized low-frequency power; HF, Normalized high-frequency power; SD1, Short-term standard deviation; SD2, Long-term standard deviation. PSS-4; Perceived Stress Scale; STAI-6, State– ,Trait Anxiety Inventory; AAQ-II, Acceptance and Action Questionnaire; SDS, Zung Self-Rating Depression Scale. bpm, Beats per minute; ms, milliseconds; %, percentage; n.u., normalized units.

**Table 4** shows significant correlations between HRV variables and psychological variables, as well as between the psychological variables themselves. Some significant correlations were observed between personality traits and HRV parameters, and more frequently, among psychological variables. Notably, conscientiousness was positively correlated with several HRV indices, whereas extraversion and psychological inflexibility showed isolated positive correlations with Max HR and SD2, respectively. Moderate correlations were found between some personality traits, specifically between anxiety, stress, depression, and psychological inflexibility.

**Table 4:** Significant correlations between HRV variables and psychological variables, as well as between the psychological variables themselves.

Correlation	Coefficient, p value
Conscientiousness - SDNN	r=0.273; p=0.046
Conscientiousness - RMSSD	r=0.350; p=0.010
Conscientiousness – pN50	r=0.361; p=0.007
Conscientiousness – HRV triangular index	r=0.285; p=0.037
Extraversion – Max HR	r=0.280; p=0.040
Psychological inflexibility (AAQII) - SD2	r=0.280; p=0.040
Extraversion – Openness	r=0.467; p<0.001
Agreeableness – Openness	r=0.425; p=0.001
Anxiety - Neuroticism	r=0.383; p=0.004
Anxiety - Psychological inflexibility (AAQII)	r=0.494; p<0.001
Anxiety - Loneliness (UCLA)	r=0.369; p=0.005
Anxiety - Stress	**r=0.624**; p<0.001
Anxiety - Depression	**r=0.516**; p<0.001
Neuroticism - Psychological inflexibility (AAQII)	r=0.300; p=0.027
Stress - Loneliness (UCLA)	r=0.273; p=0.046
Stress - Depression	**r=0.562**; p<0.001
Stress - Psychological inflexibility (AAQII)	**r=0.543**; p<0.001
Psychological inflexibility (AAQII) - Depression	r=0.397; p=0.003
Loneliness (UCLA) - Depression	r=0.483; p<0.001

Values in bold indicate moderate correlations.

In the present sample, the psychometric scales demonstrated acceptable to good internal consistency: PSS-4 (4 items, α = .767; ω = .786), STAI-6 (6 items, α = .871; ω = .878), UCLA (3 items, α = .739; ω = .803), AAQII (7 items, α = .883; ω = .884), and SDS (20 items, α = .803; ω = .835). Reliability estimates for the TIPI were lower overall, except for Neuroticism (α = .709; ω = .719). The remaining domains showed low internal consistency: Agreeableness (α = .522; ω = .527), Conscientiousness (α = .539; ω = .560), Extraversion (α = .510; ω = .515), and Openness (α = .193; ω = .210).

## Discussion

The present study characterizes the psychophysiological profile of adults who self-identify as chronically stressed. The results show a profile of psychological vulnerability, characterized by elevated levels of anxiety, depressive symptoms, and psychological inflexibility. No significant differences in resting HRV parameters were found across levels of perceived stress. Together, these findings highlight the complexity of the relationship between subjective stress experiences and autonomic regulation within a population of adults with self-perceived chronic stress (Smith et al., 2017).

Our findings are consistent with studies reporting reduced HRV associated with emotional dysfunction and lower adaptive flexibility (Laborde et al., 2017; Kim et al., 2018), as well as evidence linking decreased HRV with anxiety, depression, and difficulties in emotional regulation (McEwen, 2007). Interestingly, we found that higher conscientiousness was positively associated with several HRV indices (SDNN, RMSSD, pNN50, and HRV triangular index). This finding may suggest that individuals characterized by greater self-discipline, organization, and goal-directed behavior exhibit greater physiological flexibility and adaptive autonomic regulation. Although these associations were modest in magnitude, they are consistent with evidence linking conscientiousness to healthier behavioral patterns and more favorable health outcomes (Zahn et al., 2015).

Resting heart rate was interpreted together with HRV indices as an integrated marker of autonomic and cardiovascular regulation. In this context, higher resting heart rate—reflecting reduced HRV—may indicate increased allostatic load and could indirectly influence emotional processing and higher-order cognitive processes involved in stress regulation (Giannouli and Tsolaki, 2023).

In contrast to studies reporting marked decreases in HRV under acute or experimentally induced stress conditions (Kim et al., 2018), our sample, composed of individuals with self-perceived chronic stress, did not show statistically significant differences according to stress levels. Although only three participants reached very high stress scores, this subgroup was interpreted descriptively and did not underpin the main conclusions, which were derived from the overall sample pattern.

This discrepancy may be explained by the fact that HRV tends to decrease more sharply in response to acute demands, reflecting the immediate adaptive response of the autonomic nervous system, whereas in contexts of sustained stress, autonomic alterations are typically milder, more variable, and dependent on moderating factors such as personality, fatigue, or physiological habituation (Zacher and Rudolph, 2021; Tokarek et al., 2023). Consistent with previous findings in chronic stress populations (Kim et al., 2018), no significant differences in resting HRV were observed across perceived stress levels in the present sample. Although only three participants reached very high stress scores, this subgroup was interpreted descriptively and did not underpin the main conclusions. The observed pattern may reflect the greater heterogeneity of autonomic responses associated with chronic self-perceived stress.

The correlation pattern reinforces the interconnected nature of the emotional and physiological dimensions of stress: psychological inflexibility was positively associated with anxiety, depression, and perceived stress, whereas conscientiousness showed positive associations with several HRV indices. Together, these findings suggest that psychological vulnerability and autonomic regulation may be influenced by different psychological processes, with psychological inflexibility appearing more closely related to emotional distress and conscientiousness showing modest associations with physiological regulation.

The finding of sex differences in the spectral components of HRV (higher LF/HF in men and higher HF in women) is consistent with reviews pointing to sex-specific autonomic patterns modulated by hormonal and coping-related factors (Calderón-García et al., 2024). These results underscore the need to consider sex as a relevant variable in the interpretation of psychophysiological biomarkers.

From a neuroscientific perspective, the relative dissociation between subjective perception and vagal tone may reflect an early alteration in the communication between the prefrontal cortex and limbic structures involved in autonomic nervous system regulation (Smith et al., 2017). Clinically, identifying these profiles (high stress, high inflexibility, and mild autonomic dysfunction) may be key to preventing the chronicity of psychological distress through interventions that integrate cognitive strategies and physiological regulation techniques such as mindfulness, biofeedback, or respiratory training (Laborde et al., 2017).

Overall, the results support the notion that elevated self-perceived stress represents an early marker of psychophysiological vulnerability rather than an established autonomic dysfunction. Future studies should include longitudinal designs and stress-reactivity tests to determine whether this pattern constitutes a precursor to clinical alterations in mental or cardiovascular health.

## Conclusions

The present study provides a psychophysiological characterization of adults who self-identify as chronically stressed. The findings reveal a profile of psychological vulnerability, characterized by elevated levels of anxiety, depression, and psychological inflexibility, while no significant differences in resting HRV parameters were observed across levels of perceived stress. Together, these results suggest a complex relationship between subjective stress experiences and autonomic regulation. Furthermore, the positive associations observed between conscientiousness and several HRV indices may indicate that certain personality traits are linked to more adaptive patterns of physiological regulation. However, given the modest magnitude of these correlations, these findings should be interpreted with caution and warrant further investigation.

### Clinical applications

The results highlight the clinical relevance of integrating both subjective and physiological measures in the assessment of chronic stress. HRV analysis, combined with validated psychological questionnaires, may help characterize individuals with self-perceived chronic stress by integrating subjective psychological distress and resting autonomic markers. This integrative approach contributes to improving detection and prevention strategies in the fields of mental health and behavioral physiology, promoting personalized interventions that support both cognitive–emotional regulation and autonomic recovery.

### Limitations

The main limitations of the study include the relatively small sample size and the cross-sectional design which prevent causal inferences. Although the study was not primarily designed for categorical comparisons, the unequal distribution across stress severity levels—particularly the very small “very high stress” subgroup (n=3)—limits statistical power for subgroup analyses and requires cautious interpretation of these findings.

The absence of a non-stressed control group limits direct normative comparisons and precludes formal validation of the latent regulatory vulnerability hypothesis. Accordingly, this concept should be interpreted as an exploratory interpretative framework derived from within-sample patterns rather than as a formally tested comparative hypothesis.

Additionally, relying on self-perceived stress as an inclusion criterion may introduce a degree of subjective bias.

Regarding personality assessment, internal consistency estimates for the Ten-Item Personality Inventory (TIPI) were modest, which is consistent with prior validation studies. As previously noted, internal consistency indices such as Cronbach’s alpha are of limited interpretative value in very brief instruments designed to capture broad personality domains. In two-item scales such as the TIPI, lower alpha values are expected and reflect a deliberate trade-off to preserve content validity rather than an indication of poor measurement quality (Renau et al., 2013).

Given the home-based nature of the 7-day HRV recording protocol, environmental and contextual factors were not fully standardized. Although artifact screening and multi-day averaging were implemented to enhance stability, residual variability related to uncontrolled daily conditions cannot be entirely excluded.

Menstrual cycle phase and menopausal or perimenopausal status were not recorded or controlled. Given the known influence of hormonal fluctuations on HRV—particularly on frequency-domain indices (HF, LF, LF/HF)—this biological variability may have contributed to variability in spectral measures.

Future studies should employ longitudinal and experimental designs with larger samples to allow a more precise examination of the causal pathways linking psychological traits and autonomic regulation in the context of chronic stress, incorporating matched control groups and systematic control of relevant biological variables.

## Data Availability

The original contributions presented in the study are included in the article/supplementary material. Further inquiries can be directed to the corresponding author.

## References

[B1] Altungy P. Liébana S. Sánchez-Marqueses J. M. Sanz-García A. García-Vera M. P. Sanz J. (2023). Psychometric properties of the Anxiety Sensitivity Index-3 (ASI-3) in Spanish population. Psicothema 35, 300–309. doi: 10.7334/psicothema2022.237 37493153

[B2] Benarroch E. E. (1993). The central autonomic network: Functional organization, dysfunction, and perspective. Mayo Clin. Proc. 68, 988–1001. doi: 10.1016/S0025-6196(12)62272-1 8412366

[B3] Calderón-García A. Álvarez-Gallardo E. Belinchón-deMiguel P. Clemente-Suárez V. J. (2024). Gender differences in autonomic and psychological stress responses among educators: a heart rate variability and psychological assessment study. Front. Psychol. 15, 2024. Available online at: https://www.frontiersin.org/journals/psychology/articles/10.3389/fpsyg.2024.1422709 (Accessed July 8, 2026). 10.3389/fpsyg.2024.1422709 PMC11494829 39439753

[B4] Chand K. Chandra S. Dutt V. (2024). A comprehensive evaluation of linear and non-linear HRV parameters between paced breathing and stressful mental state. Heliyon 10, e32195. doi: 10.1016/j.heliyon.2024.e32195 38873683 PMC11170182

[B5] Clemente-Suárez V. J. Ruisoto-Palomera P. (2020). Effects of the psychophysiological stress response in human behavior. Physiol. Behav. 214, 112761. doi: 10.1016/j.physbeh.2019.112761 31785268

[B6] Cohen S. Janicki-Deverts D. Miller G. E. (2007). Psychological stress and disease. JAMA 298, 1685–1687. doi: 10.1001/jama.298.14.1685 17925521

[B7] Franklin B. A. Rusia A. Haskin-Popp C. Tawney A. (2021). Chronic stress, exercise and cardiovascular disease: Placing the benefits and risks of physical activity into perspective. Int. J. Environ. Res. Public Health 18, 9922. doi: 10.3390/ijerph18189922 34574843 PMC8471640

[B8] Gallup (2022). Gallup Global Emotions. Available online at: https://www.gallup.com/analytics/349280/gallup-global-emotions-report.aspx (Accessed July 8, 2026).

[B9] Giannouli V. (2025). “ The impact of stressful life events in alzheimer’s disease,” in Handbook of the Behavior and Psychology of Disease. Eds. Martin C. R. Preedy V. R. Patel V. B. Rajendram R. ( Springer Nature Switzerland, Cham), 1251–1266. doi: 10.1007/978-3-031-73363-5_55

[B10] Giannouli V. Tsolaki M. (2023). Stressful life events, general cognitive performance, and financial capacity in healthy older adults and Alzheimer’s disease patients. Neuropsychiatrie 37, 76–79. doi: 10.1007/s40211-022-00451-y 36600106 PMC10238300

[B11] Goroso D. G. Watanabe W. T. Napoleone F. Da Silva D. P. Salinet J. L. Da Silva R. R. . (2021). Remote monitoring of heart rate variability for obese children. Biomed. Signal Process. Control 66, 102453. doi: 10.1016/j.bspc.2021.102453 38826717

[B12] Hayano J. Yuda E. (2019). Pitfalls of assessment of autonomic function by heart rate variability. J. Physiol. Anthropol. 38, 1–28. doi: 10.1186/s40101-019-0193-2 30867063 PMC6416928

[B13] Health, M (2022). Brain Health and Substance Use (MSD. Available online at: https://www.who.int/publications/i/item/9789240050860 (Accessed July 8, 2026).

[B14] Hernando A. Lázaro J. Gil E. Arza A. Garzón J. M. López-Antón R. . (2016). Inclusion of respiratory frequency information in heart rate variability analysis for stress assessment. IEEE J. Biomed. Health Inform. 20, 1016–1025. doi: 10.1109/JBHI.2016.2553578 27093713

[B15] Hughes M. E. Waite L. J. Hawkley L. C. Cacioppo J. T. (2004). A short scale for measuring loneliness in large surveys: Results from two population-based studies. Res. Aging 26, 655–674. doi: 10.1177/0164027504268574 18504506 PMC2394670

[B16] Kiecolt-Glaser J. K. Fagundes C. P. Andridge R. Peng J. Malarkey W. B. Habash D. . (2017). Depression, daily stressors and inflammatory responses to high-fat meals: When stress overrides healthier food choices. Mol. Psychiatry 22, 476–482. doi: 10.1038/mp.2016.149 27646264 PMC5508550

[B17] Kim H. G. Cheon E. J. Bai D. S. Lee Y. H. Koo B. H. (2018). Stress and heart rate variability: A meta-analysis and review of the literature. Psychiatry Investig. 15, 235–245. doi: 10.30773/pi.2017.08.17 29486547 PMC5900369

[B18] Laborde S. Mosley E. Thayer J. F. (2017). Heart rate variability and cardiac vagal tone in psychophysiological research: Recommendations for experiment planning, data analysis, and data reporting. Front. Psychol. 8. doi: 10.3389/fpsyg.2017.00213 28265249 PMC5316555

[B19] Lee R. S. (2022). “ The physiology of stress and the human body’s response to stress,” in Epigenetics of Stress and Stress Disorders 31. Ed. Youssef E. N. A. . Academic Press. 1–18. doi: 10.1016/B978-0-12-823039-8.00017-4

[B20] Lehrer P. (2013). How does heart rate variability biofeedback work? Resonance, the baroreflex, and other mechanisms. Biofeedback 41, 26–31. doi: 10.5298/1081-5937-41.1.02

[B21] Madison A. A. Andridge R. Shrout M. R. Renna M. E. Bennett J. M. Jaremka L. M. . (2022). Frequent interpersonal stress and inflammatory reactivity predict increases in depressive symptoms: two tests of the social signal transduction theory of depression. Psychol. Sci. 33 (1), 152–164. doi: 10.1177/09567976211031225 34932407 PMC8985224

[B22] Malik M. (1996). Heart rate variability: standards of measurement, physiological interpretation, and clinical use task force of the European society of cardiology and the North American society for pacing and electrophysiology. Ann. Noninvasive Electrocardiol. 1, 151–181. doi: 10.1111/j.1542-474X.1996.tb00275.x 40046247

[B23] Manga D. Ramos F. Morán C. (2004). The Spanish norms of the NEO five-factor inventory: new data and analyses for its improvement. Int. J. Psychol. Psychol. Ther. 24, 639–648.

[B24] Marteau T. M. B H. (2020). The development of a shortened six-item version of the Spielberger State-Trait Anxiety Inventory (STAI) state scale. Br. J. Clin. Psychol. 59, 276–288. doi: 10.1111/bjc.12243 1393159

[B25] Massaro S. Pecchia L. (2019). Heart rate variability (HRV) analysis: A methodology for organizational neuroscience. Organ. Res. Methods 22, 354–393. doi: 10.1177/1094428116681072

[B26] McEwen B. S. (2007). Physiology and neurobiology of stress and adaptation: central role of the brain. Physiol. Rev. 87, 873–904. doi: 10.1152/physrev.00041.2006 17615391

[B27] Mukaka M. M. (2012). Statistics corner: a guide to the proper use of correlation coefficient in medical research. Malawi Med. J. 24, 69–71. 23638278 PMC3576830

[B28] Nunan D. Sandercock G. R. H. Brodie D. A. (2010). A quantitative systematic review of normal values for short-term heart rate variability in healthy adults. PACE Pacing Clin. Electrophysiol. 33, 1407–1417. doi: 10.1111/j.1540-8159.2010.02841.x 20663071

[B29] Pérez-Alcalde A. I. Galán-del-Río F. Fernández-Rodríguez F. J. de la Plaza San Frutos M. García-Arrabé M. Giménez M.-J. . (2024). The effects of a single vagus nerve’s neurodynamics on heart rate variability in chronic stress: A randomized controlled trial. Sens. 14248220 24, 6874–6889. doi: 10.3390/s24216874 39517768 PMC11548125

[B30] Porges S. W. (2011). The polyvagal theory: neurophysiological foundations of emotions, attachment, communication, and self-regulation Norton Series on Interpersonal Neurobiology (New York, NY: WW Norton & Company). Available online at: http://ci.nii.ac.jp/ncid/BB05274933 (Accessed July 8, 2026).

[B10000] Porges S. W (2022). Polyvagal theory: A science of safety. Front. Integr. Neurosci. 16, 1–12. doi: 10.3389/fnint.2022.871227 PMC9131189 35645742

[B10001] Renau V. Oberst U. Gosling S. D. Rusiñol J. Chamarro A. (2013). Translation and validation of the Ten-Item Personality Inventory into Spanish and Catalan. Aloma: Revista de Psicologia, Ciències de l'Educació i de l'Esport 31 (2), 85–97.

[B31] Rodríguez-Jiménez R.-M. Carmona M. García-Merino S. Díaz-Ureña G. Lara Bercial P. J. (2022). Embodied learning for well-being, self-awareness, and stress regulation: A randomized trial with engineering students using a mixed-method approach. Educ. Sci. 12, 111. doi: 10.3390/educsci12020111 30654563

[B32] Ruiz F. J. Suárez-Falcón J. C. Cárdenas-Sierra S. Durán Y. Guerrero K. Riaño-Hernández D. (2016). Psychometric properties of the acceptance and action questionnaire–II in Colombia. Psychol. Rec. 66, 429–437. doi: 10.1007/s40732-016-0183-2 30311153

[B33] Rusanov V. B. Fomina E. V. Orlov O. I. (2022). Does heart rate variability reflect brain plasticity as a likely mechanism of adaptation to space missions? Front. Space Technol. 3. doi: 10.3389/frspt.2022.998610

[B34] Santos G. S. P. Costa A. C. Picoli C. C. Rocha B. G. S. Sulaiman S. O. Radicchi D. C. . (2022). Sympathetic nerve-adipocyte interactions in response to acute stress. J. Mol. Med. 100, 151–165. doi: 10.1007/s00109-021-02157-0 34735579 PMC8567732

[B35] Schneider M. Schwerdtfeger A. (2020). Autonomic dysfunction in posttraumatic stress disorder indexed by heart rate variability: A meta-analysis. Psychol. Med. 50, 1937–1948. doi: 10.1017/S003329172000207X 32854795 PMC7525781

[B36] Schubert C. Lambertz M. Nelesen R. A. Bardwell W. Choi J.-B. Dimsdale J. E. (2009). Effects of stress on heart rate complexity—A comparison between short-term and chronic stress. Biol. Psychol. 80, 325–332. doi: 10.1016/j.biopsycho.2008.11.005 19100813 PMC2653595

[B37] Shaffer F. Ginsberg J. P. (2017). An overview of heart rate variability metrics and norms. Front. Public Health 5. doi: 10.3389/fpubh.2017.00258 29034226 PMC5624990

[B38] Shaffer F. Meehan Z. M. (2020). A practical guide to resonance frequency assessment for heart rate variability biofeedback. Front. Neurosci. 14. doi: 10.3389/fnins.2020.570400 33117119 PMC7578229

[B39] Sharma N. Gedeon T. (2012). Objective measures, sensors and computational techniques for stress recognition and classification: A survey. Comput. Methods Programs Biomed. 108, 1287–1303. doi: 10.1016/j.cmpb.2012.07.003 22921417

[B40] Smith R. Thayer J. F. Khalsa S. S. Lane R. D. (2017). The hierarchical basis of neurovisceral integration. Neurosci. Biobehav. Rev. 75, 274–296. doi: 10.1016/j.neubiorev.2017.02.003 28188890

[B41] Teodorescu V. Nanea T. (2014). Măsurătorile standard, interpretarea fi ziologică şi clinică a variabilităţii ritmului cardiac. Perspect. Clin. Postinfarct Romanian J. Med. Pract. 9, 148–153.

[B42] Thayer J. F. Åhs F. Fredrikson M. Sollers J. J. Wager T. D. (2011). A meta-analysis of heart rate variability and neuroimaging studies: Implications for heart rate variability as a marker of stress and health. Neurosci. Biobehav. Rev. 36, 747–756. doi: 10.1016/j.neubiorev.2011.11.009 22178086

[B43] Thørrisen M. M. Sadeghi T. (2023). The Ten-Item Personality Inventory (TIPI): a scoping review of versions, translations and psychometric properties. Front. Psychol. 14. doi: 10.3389/fpsyg.2023.1202953 37434881 PMC10330951

[B44] Tokarek J. Kapuścik A. Kućmierz J. Kowalczyk E. Karbownik M. S. (2023). Personality traits and health-related behaviors in medical students facing a stressful event. Front. Public Health 11, 1256883. doi: 10.3389/fpubh.2023.1256883 37900039 PMC10602886

[B45] Veloza L. Jiménez C. Quiñones D. Polanía F. Pachón-Valero L. C. Rodríguez-Triviño C. Y. (2019). Variabilidad de la frecuencia cardiaca como factor predictor de las enfermedades cardiovasculares. Rev. Colomb. Cardiol. 26, 205–210. doi: 10.1016/j.rccar.2019.01.006 38826717

[B46] Vigo D. Thornicroft G. Atun R. (2016). Estimating the true global burden of mental illness. Lancet Psychiatry 3, 171–178. doi: 10.1016/S2215-0366(15)00505-2 26851330

[B47] Warttig S. L. Forshaw M. J. South J. White A. K. (2013). New, normative, English-sample data for the Short Form Perceived Stress Scale (PSS-4. J. Health Psychol. 18, 1617–1628. doi: 10.1177/1359105313508346 24155195

[B48] Zacher H. Rudolph C. W. (2021). Big Five traits as predictors of perceived stressfulness of the COVID-19 pandemic. Personal. Individ. Differ. 175, 110694. doi: 10.1016/j.paid.2021.110694 33531723 PMC7843115

[B49] Zahn R. Lythe K. E. Gethin J. A. Green S. Deakin J. F. W. Young A. H. . (2015). The role of self-blame and worthlessness in the psychopathology of major depressive disorder. J. Affect. Disord. 186, 337–341. doi: 10.1016/j.jad.2015.08.001 26277271 PMC4573463

[B50] Zung W. W. K. (1965). A self-rating depression scale. Arch. Gen. Psychiatry 12, 63–70. doi: 10.1037/t04095-000 14221692

